# Int6 reduction activates stromal fibroblasts to enhance transforming activity in breast epithelial cells

**DOI:** 10.1186/s13578-015-0001-6

**Published:** 2015-03-08

**Authors:** Jinfeng Suo, Daniel Medina, Sabrina Herrera, Ze-Yi Zheng, Lei Jin, Gary C Chamness, Alejandro Contreras, Carolina Gutierrez, Susan Hilsenbeck, Arzu Umar, John A Foekens, Samir Hanash, Rachel Schiff, Xiang H-F Zhang, Eric C Chang

**Affiliations:** Department of Clinical Cancer Prevention, The University of Texas MD Anderson Cancer Center, Houston, 77030 TX USA; Department of Molecular and Cellular Biology and Lester and Sue Smith Breast Center, Baylor College of Medicine, Houston, 77030 TX USA; Department of Medical Oncology, Erasmus MC Cancer Institute, Erasmus University Medical Center, Rotterdam, Netherlands

**Keywords:** Translation initiation factor, HMFs, SMA, SDF-1, CXCR4, CAF, Stroma, Tumor microenvironment, eIF3e

## Abstract

**Background:**

The *INT6* gene was first discovered as a site of integration in mouse mammary tumors by the mouse mammary tumor virus; however, *INT6*’s role in the development of human breast cancer remains largely unknown. By gene silencing, we have previously shown that repressing *INT6* promotes transforming activity in untransformed human mammary epithelial cells. In the present study, guided by microarray data of human tumors, we have discovered a role of Int6 in stromal fibroblasts.

**Results:**

We searched microarray databases of human tumors to assess Int6’s role in breast cancer. While *INT6* expression levels, as expected, were lower in breast tumors than in adjacent normal breast tissue samples, *INT6* expression levels were also substantially lower in tumor stroma. By immunohistochemistry, we determined that the low levels of *INT6* mRNA observed in the microarray databases most likely occurs in stromal fibroblasts, because far fewer fibroblasts in the tumor tissue showed detectable levels of the Int6 protein. To directly investigate the effects of Int6 repression on fibroblasts, we silenced *INT6* expression in immortalized human mammary fibroblasts (HMFs). When these *INT6*-repressed HMFs were co-cultured with breast cancer cells, the abilities of the latter to form colonies in soft agar and to invade were enhanced. We analyzed *INT6*-repressed HMFs and found an increase in the levels of a key carcinoma-associated fibroblast (CAF) marker, smooth muscle actin. Furthermore, like CAFs, these *INT6*-repressed HMFs secreted more stromal cell–derived factor 1 (SDF-1), and the addition of an SDF-1 antagonist attenuated the *INT6*-repressed HMFs’ ability to enhance soft agar colony formation when co-cultured with cancer cells. These *INT6*-repressed HMFs also expressed high levels of mesenchymal markers such as vimentin and N-cadherin. Intriguingly, when mesenchymal stem cells (MSCs) were induced to form CAFs, Int6 levels were reduced.

**Conclusion:**

These data suggest that besides enhancing transforming activity in epithelial cells, *INT6* repression can also induce fibroblasts, and possibly MSCs as well, via mesenchymal-mesenchymal transitions to promote the formation of CAFs, leading to a proinvasive microenvironment for tumorigenesis.

## Background

The mammalian *INT6* gene was first discovered in a genetic screen using the mouse mammary tumor virus (MMTV) to isolate the *INT* genes that mediate breast tumorigenesis [1]. MMTV insertions into the *INT6* gene apparently cause the expression of C-terminally truncated Int6 proteins (Int6ΔC), which when ectopically overexpressed induce cell transformation and tumor formation in mouse models [2,3]. We and others [4-6] have identified an *INT6* ortholog in the fission yeast *Schizosaccharomyces pombe.* While full-length human Int6 rescues the *S. pombe* Int6-null phenotype, Int6∆C does not [7]. Therefore it is highly probable that Int6∆C acts in a dominant-negative fashion to promote tumor formation in mouse mammary glands.

In human breast cancer, we and others have examined several breast cancer cell lines but found no evidence of Int6∆C expression [3,8]. However, several earlier studies have shown lower levels of *INT6* expression in breast cancer than normal tissues supporting the possibility that Int6 acts as a tumor suppressor [9-11]. Int6 is also known as eIF3e, a component of the eukaryote translation initiation factor [12]; in addition, Int6 has been shown to control nonsense-mediated mRNA decay [13]. These data suggest that Int6 can affect efficient translation. Using *S. pombe,* we revealed a new activity of Int6 — regulation of the 26S proteasome [7], abnormality in which increases levels of cyclin and securin, leading to abnormal mitosis and chromosome instability. Furthermore, using mass spectrometry to identify all Int6-interacting proteins, we found that Int6 is in a supercomplex—which we named the translasome—that contains all the components needed for translation as well as proteasome subunits [14]. This discovery led to the hypothesis that Int6 can fine-tune levels of key regulatory proteins by coordinating protein synthesis and protein degradation within the translasome. As such, Int6 reduction may induce tumor formation in breast epithelial cells by causing a net increase in the levels of proteins that promote tumorigenesis. In support of this hypothesis, we and others have recently shown that repressing *INT6* expression in normal mammary epithelial cells induces a transforming phenotype, which correlates with the stabilization of a potent oncoprotein, Src3/AIB1, and altered translation of the ubiquitin genes as well as of genes controlling the epithelial-mesenchymal transition (EMT) [8,15].

While solid tumors are mostly derived from epithelial cells, increasing evidence suggests that the tumor microenvironment can play important roles in influencing tumor progression. Within the tumor microenvironment, as much as half the mass of the tumor microenvironment is made of stromal cells [16]. A key component of the stroma is the fibroblast. Fibroblasts isolated from solid tumors (called carcinoma-associated fibroblasts, CAFs), when co-transplanted with carcinoma cells, can strongly promote *in vivo* tumor growth as well as angiogenesis and metastasis [17-19]. Eliminating CAFs has been reported to suppress spontaneous metastasis and to enhance the anti-metastatic effects of chemotherapy in mouse breast cancer models [20].

CAFs from invasive human breast carcinomas appear to control tumor cells by secreting a number of stromal cell-derived factors, the chief of which is stromal cell–derived factor 1 (SDF-1), also called CXCL12 [18]. SDF-1 signals via its cognate receptor, CXCR4. When CXCR4 is expressed on the surface of carcinoma cells, CAFs can directly enhance the proliferation of these cells via an SDF-1/CXCR4 paracrine loop. The most potent CAFs for promoting tumor progression appear to be a fraction called myofibroblasts, which are marked by the expression of smooth muscle actin (α-SMA). High levels of myofibroblasts in CAFs correlate with robust growth of co-transplanted xenografted human tumors [21]; furthermore, in human breast cancer patients, high α-SMA levels in the tumor stroma correlate with poor clinical outcomes [22,23].

In this study, guided by analyses of tumor microarray databases, we came upon the surprising finding that, while *INT6* mRNA levels are as expected lower in breast cancer than in normal tissue, *INT6* mRNA levels are also very low in the tumor stroma. By immunohistochemistry (IHC) staining, we determined that Int6 levels are low in the fibroblasts in tumor stroma. We went on to show that *INT6* repression can induce normal mammary fibroblasts to act like CAFs, apparently by activating a mesenchymal-mesenchymal transition (MMT). Our results suggest that the reduction of Int6 can promote breast tumor formation not only by activating oncogenic pathways in epithelial cells but also by inducing a CAF-like activity in the stromal fibroblasts.

## Results

### Int6 is reduced in the fibroblasts in human breast cancer

**Figure 1:**
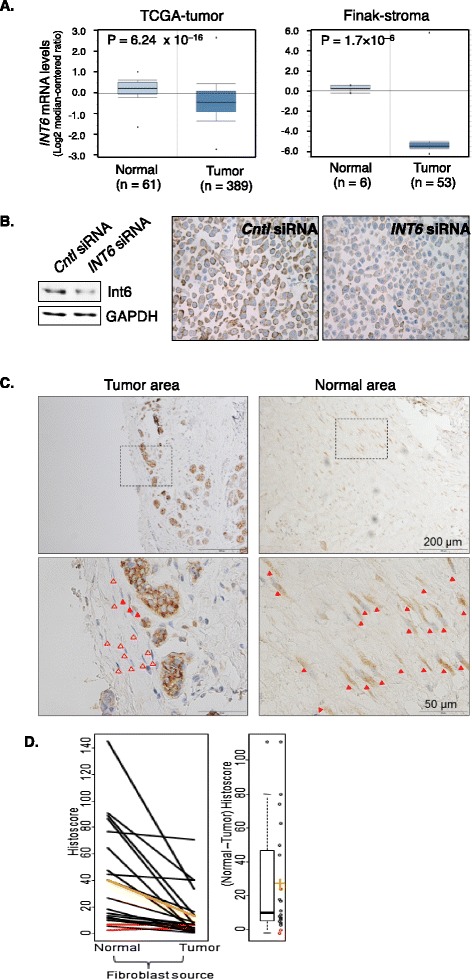
**Reduction of Int6 in the fibroblasts in human breast tumors. (A)** Left: gene expression data from the breast cancer TCGA project were directly exported from Oncomine, in which mRNA levels of *INT6* in normal breast tissue and invasive ductal carcinomas were compared. *INT6* mRNA levels were reported to be 50% lower in the latter. Right: stroma gene expression data in the Finak study available in Oncomine were analyzed to show that *INT6* mRNA levels were approximately 42 times higher in the tissue surrounding the normal adjacent ducts than in the stroma in the tumor. **(B)** Control or *INT6*-repressed MCF7 cells were analyzed by Western blot (left) or IHC (right) by an anti-Int6 antibody. We note that agreeing with our previous finding using GFP-tagging [26], Int6 is mainly cytoplasmic. **(C)** This is a typical IHC experiment examining fibroblasts in the adjacent normal and tumor region from the same human tumor sample. The top pictures were captured using a 10× objective, and one area in each was then examined by a 40× objective to reveal more details. Closed and open arrowheads mark Int6-positive vs. Int6-negative fibroblasts. **(D)** The histoscore differences between the normal and tumor regions were compared by the Wilcoxon signed rank test. While there was no difference in Int6 intensity between the normal and tumor regions (p = 0.66), a much lower percentage of Int6-positive fibroblasts was found in the tumor. As a result, all but two samples (marked red) show lower values for tumor fibroblasts. The mean normal and tumor histoscores are marked orange. On the right is a boxplot of the differences between normal and tumor histoscores (individual values shown as circles, mean difference shown in orange). Mean difference ± SEM = 27.0 ± 7.2 (p < 10^−3^, Wilcoxon signed rank test).

To determine whether *INT6* may act as a tumor suppressor for breast cancer, we searched Oncomine for gene expression changes by focusing on studies in which normal and tumor tissues were compared. *INT6* levels were found to be significantly higher in normal tissues compared with invasive breast tumors and premalignant ductal carcinoma in situ (DCIS), according to data from the TCGA project (Figure 1A, left) [24] and Curtis et al. (data not shown) [25], respectively.

Most intriguingly, data from studies of gene expression in the stroma showed even more drastic differences between *INT6* expression levels in tumors and normal tissues. For example, data from Finak et al. [9] showed that *INT6* expression levels were about 42 times lower in the stroma of invasive carcinoma than in matched adjacent normal tissue (Figure 1A, right). Likewise, data from Ma et al. [27] showed that *INT6* expression levels were lower in the stroma of invasive carcinoma and also in DCIS than in normal tissue (data not shown).

To further examine whether Int6 is downregulated in tumor stroma at the protein level, we established an IHC protocol by first examining Int6 levels in parental and *INT6*-silenced MCF7 cells. As shown in Figure 1B, both Western blot and IHC showed a 50% reduction in Int6 levels in the silenced cells, suggesting that our IHC protocol could detect Int6 with a high degree of specificity. We then stained paraffin-embedded tumor tissues with hematoxylin and eosin (H&E) to select 20 samples that contained stroma at least 2 mm away from the tumor, which we and Finak at al. defined as normal stroma [9]. In these normal stromal regions, we readily detected fibroblasts that stained strongly for Int6 (Figure 1C). In contrast, such Int6-positive fibroblasts were rare in the tissue proximal (<2 mm) to the carcinoma cells. We could also detect Int6 in plasma cells, but the levels did not differ substantially between normal and tumor tissues. We scored the Int6 intensity and calculated the histoscore differences between the normal and tumor regions (Figure 1D). While there was not a dramatic difference in Int6 intensity between the normal and tumor regions (p = 0.66), the fraction of fibroblasts that were Int6-positive in the normal region was much higher (p < 0.001), leading to higher histoscores (p = 0.0014). These data agree with the concept that Int6 reduction in stromal fibroblasts (in addition to epithelial cells) may promote breast tumorigenesis.

### Int6 reduction in normal human mammary fibroblasts induces CAF-like properties

**Figure 2:**
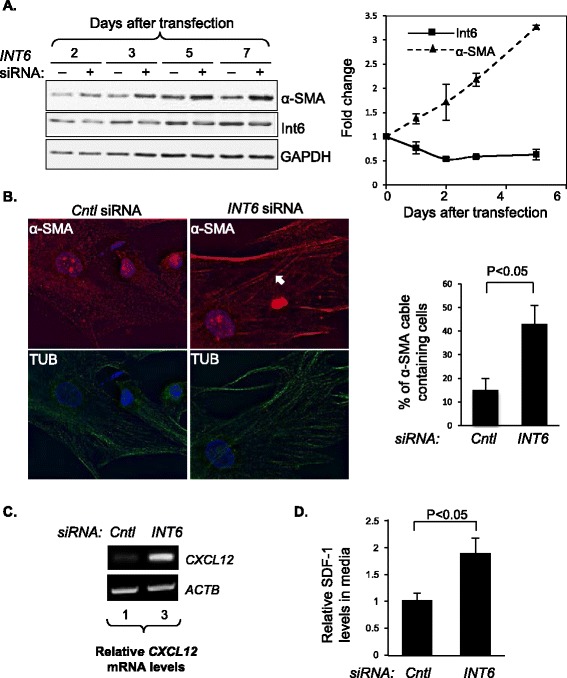
**Reduction of Int6 induces CAF-like properties in normal human mammary fibroblasts. (A)** HMFs were transfected with control or anti-*INT6* siRNA. On the left, protein samples were analyzed over time by Western blots using antibodies against α-SMA and Int6. GAPDH was the loading control. On the right, a quantification of Western blots from three separate experiments was graphed. **(B)** HMFs were treated with control or anti-*INT6* siRNA for 5 days and then immunostained with antibodies against α-SMA (red) and tubulins (green), and counter-stained by DAPI (blue) to mark the nuclei. Cells containing α-SMA cables were counted (n = 100 cells). White arrow indicates a typical α-SMA cable in a cell. Quantification of cells containing α-SMA cables is shown on the right. **(C)** On the left, semiquantitative RT-PCR was performed to measure *CXCL12* (encoding SDF-1) mRNA levels in parental and Int6-repressed HMFs. *CXCL12* mRNA levels were normalized to those of *ACTB,* and the normalized *CXCL12* mRNA levels in the control were set to 1. **(D)** The growth media of the cells from panel C were analyzed by ELISA to measure SDF-1 levels 7 days after seeding. SDF-1 levels in the control were set to 1 (n = 3 separate experiments).

To investigate whether Int6 reduction in fibroblasts can induce CAF-like activities, we repressed *INT6* expression using siRNA in h-TERT-immortalized normal human mammary fibroblasts (HMFs) and then measured a key CAF marker, α-SMA. As shown in Figure 2A, repressing *INT6* steadily increased α-SMA levels over a 5-day period after gene silencing. To determine whether the increase in α-SMA levels resulted in more efficient formation of α-SMA cables in the cells, we performed immunostaining. As shown in Figure 2B, greater than 2.5 times more *INT6-*repressed HMFs contained α-SMA cables.

CAFs are also known to influence epithelial cells by paracrine signaling through the secretion of SDF-1. To examine whether *INT6* repression induces the expression of *CXCL12*, which encodes SDF-1, in stromal fibroblast cells, we examined *INT6*-repressed HMFs and found that their mRNA levels increased over a 7-day period (Figure 2C). Levels of secreted SDF-1 also increased in the medium of *INT6*-repressed HMFs (Figure 2D). These data collectively suggest that CAFs can be derived from fibroblasts when Int6 levels are downregulated.

### *INT6*-silenced HMFs enhance transforming activities in breast cancer cells

**Figure 3:**
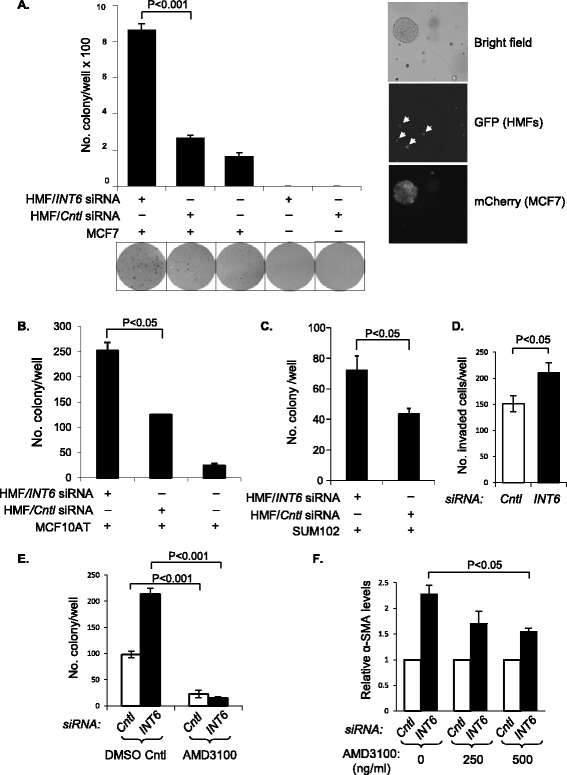
***INT6*** **-repressed HMFs enhance transformation phenotypes of breast cancer cells. (A)** HMFs were transfected with either control siRNA or anti-*INT6* siRNA. A fraction of these cells was analyzed by Western blot to confirm the reduction in Int6 and α-SMA levels (data not shown). The rest were mixed with MCF7 cells before seeding in triplicate (n = 3). The colonies in each well (a representative area from each is shown below the graph) were counted after 15 days. We note that HMFs seeded alone do not form colonies in soft agar (column 4 and column 5). To confirm that the emerged colonies are of cancer cells, we tagged MCF7 cells with mCherry and found that all colonies were enriched with mCherry-positive cells (right). The HMFs were already tagged by GFP (marked by white arrow heads) [31], but we did not detect large colonies full of GFP-positive cells. **(B)** MCF10AT cells were examined similarly as in panel **A**, except that we did not include the HMF-alone control. **(C)** SUM102 cells were examined similarly as in panel **B**. **(D)** MCF7 cells were loaded in an invasion chamber and submerged in conditioned medium from control or *INT6*-repressed HMFs. Invaded cancer cells from five different areas (n = 5) on each insert membrane were counted. **(E)** Normal and *INT6*-repressed HMFs were mixed with MCF7 cells and seeded in triplicate (n = 3) into soft agar with or without AMD3100 (500 ng/mL). Colonies were counted after 15 days. **(F)** *INT6*-repressed HMFs were co-cultured with MCF7 cells for 4 days before AMD3100 was added (500 ng/mL). After 24 hours, the α-SMA levels in HMFs were measured by Western blot. α-SMA levels, normalized by the loading control GAPDH from the control cells, were set as 1 (n = 4 separate experiments).

To determine whether *INT6*-repressed HMFs can functionally affect transforming phenotypes of breast cancer cells, we first analyzed colony formation in soft agar with or without co-cultured HMFs. As shown in Figure 3A, while parental HMFs can weakly enhance colony formation by MCF7 cells in soft agar, when *INT6* was repressed in HMFs, colony formation increased 5 fold. We have obtained similar results with a variant of MCF7 cells [21] (data not shown). To further investigate this concept, we examined two preinvasive cell lines, MCF10AT [28] (Figure 3B) and SUM102 cells [29,30] (Figure 3C), and found that *INT6*-repressed HMFs can also readily enhance colony formation in soft agar. Next, we analyzed cell invasiveness using a Matrigel-coated invasion chamber and found that *INT6*-repressed HMFs can more efficiently attract MCF7 cells across the Matrigel, suggesting that the invasiveness of cancer cells can be enhanced by *INT6*-repressed HMFs (Figure 3D). To determine whether SDF-1 is a key signaling molecule secreted by *INT6*-repressed HMFs to influence transforming activities in cancer cells, we added the SDF-1 receptor antagonist AMD3100 and found that HMF-induced colony formation in soft agar was greatly reduced (Figure 3E), with a concurrent reduction of the CAF marker α-SMA (Figure 3F). Collectively, these results demonstrate that *INT6*-repressed HMFs can function like CAFs to enhance transforming phenotypes of several breast cancer cell lines.

### Int6 reduction may promote CAF formation via a MMT mechanism

**Figure 4:**
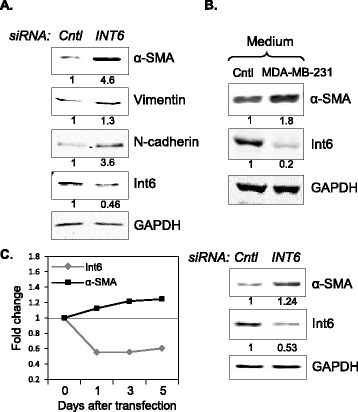
***INT6*** **repression may induce MMT. (A)** HMFs were transfected with control or anti-*INT6* siRNA for 3 days, and their lysates were analyzed by Western blots to detect the indicated proteins. Shown here is a representative experiment in which levels of mesenchymal markers increased when *INT6* was silenced. Protein levels normalized to GAPDH in the control cells were set to 1. **(B)** Human MSCs were incubated in conditioned medium from MDA-MD-231 cells for 10 days before they were analyzed by Western blot to detect α-SMA and Int6 as in panel **A**. **(C)** Protein levels of α-SMA and Int6 in control or *INT6*-repressed MSCs were measured by Western blot over time (left). The data from day 5 are shown as an example (right).

The mechanism(s) by which CAFs are generated are poorly understood. However, Int6 reduction has been shown to induce EMT [15], suggesting that Int6 reduction may induce more mesenchymal traits in the cell. We thus investigated whether *INT6* repression promotes CAF-like properties by inducing mesenchymal traits in HMFs. As shown in Figure 4A, when *INT6* expression was silenced, protein levels of two mesenchymal markers, vimentin and N-cadherin, showed an average increase of 1.5 and 2.5 times respectively, in two experiments.

Mesenchymal stem cells (MSCs) have been shown to produce CAF-like cells, presumably by a trans-differentiation process called MMT, and these cells can be detected when co-cultured with cancer cells [32,33]. As shown in Figure 4B, as MSCs were so induced to produce CAF-like phenotype, we found that Int6 levels were decreased in MSCs. To directly investigate whether *INT6* repression in MSCs can also induce CAF-like activity, we repressed *INT6* and found that α-SMA levels were indeed elevated in the MSCs (Figure 4C).

## Discussion

In this study, we show that *INT6* expression is reduced not only in tumor cells but also in the stroma. Our IHC data illustrate that this reduction is mainly due to low levels of Int6 in fibroblasts. To directly investigate that Int6 reduction in fibroblasts is functionally relevant to tumorigenesis, we repressed *INT6* in an immortalized HMF cell line and found that these cells can promote anchorage-independent growth and invasion in co-cultured breast cancer cells. These HMFs show CAF-like phenotypes—in particular, their SDF-1 secretion is increased; SDF-1 repression can substantially retard transformation. These results support the model that Int6 reduction in fibroblasts can induce them to a CAF-like state, creating a proinvasive tumor microenviro nment.

While the presence of CAFs is widely accepted as a key factor for promoting tumorigenesis, the origin of these CAFs remains largely unclear. One key area of focus is the stem cells in various stromal components (e.g., endothelial vessels, fat) that appear to differentiate into CAFs under the influence of cancer cells. In addition, breast cancer frequently metastasizes to the bone, and it has been shown that MSCs in the bone can be reprogrammed to form CAFs, thus creating a microenvironment favorable for bone metastasis [34]. In addition to these possibilities, data from this study and others [21] support the concept that CAFs can also be derived from fibroblasts themselves. We further speculate that Int6 reduction may be one of the common steps leading to the formation of CAFs, because when MSCs were induced by co-cultured cancer cells to form CAFs, Int6 levels were reduced, and a direct reduction of Int6 in MSCs can induce a CAF-like phenotype.

While our data are consistent with the model that Int6 reduction can induce MMT, the molecular mechanism for how this occurs is complex and not resolved in this study. Int6 is also known as eIF3e, a component of the translation initiation factor 3. We and others have shown that Int6 is not essential for translation; rather, it can selectively control the translation of a subset of genes [8,35]. In addition, we and others have found that Int6 can control the stability of key regulatory proteins by interacting with 26S proteasomes. Collectively, these data suggest that Int6 can control levels of key regulatory proteins via a combined and coordinated alteration of translation and proteolysis to globally reprogram cellular activity in an efficient manner. Targeting translation or inhibiting proteasomes have already emerged as powerful clinical approaches to treat cancers [36,37].

## Conclusions

We have uncovered a previously unknown tumor suppressor activity for Int6, namely, that its loss can stimulate the formation of stromal fibroblasts to a CAF-like state to enhance transforming activities in breast epithelial cells. Our data support a model in which CAFs can be generated not only from MSCs but also from fibroblasts themselves when Int6 activity is attenuated. It is possible that Int6 inactivation promotes a mesenchymal state by altering the translation and/or stability of a subset of key regulatory proteins.

## Methods

### Cells and general culture conditions

MCF7, MDA-MB-231, MCF7-ras, MCF10AT and SUM102 cells were obtained from the American Type Culture Collection. HMFs (a kind gift from Charlotte Kuperwasser, Tufts University), human MSCs, and MCF10AT cells were cultured as previously described [8,31,38]. MCF7, MDA-MB-231, MCF7-ras and SUM102 cells were cultured in Dulbecco’s Modified Eagle Medium (GIBCO) supplemented with 5% fetal bovine serum, 100 IU/mL penicillin, 0.1 mg/mL streptomycin, and 2 mM glutamine (GIBCO). For co-culturing HMFs with cancer cells, the former were seeded first to allow for attachment before a 0.4-μm Millicell insert (EMD Millipore) was placed on top of them. Cancer cells were then loaded into the insert and grown on the membrane in the insert. These thus shared the same culture medium but were kept physically separated. SDF-1 in the culture media was detected by an enzyme-linked immunosorbent assay (ELISA) kit from R&D Systems. The siRNA used to knock down *INT6* expression was thoroughly tested as previously described by us and by others [8,39].

### Assays for anchorage-independent growth and invasion

The cells were seeded and grown in soft agar as described previously [8], except that the cancer cells were mixed with HMFs at a ratio of 1:1. Ten thousand cells were seeded in each well of a 6-well plate. To block SDF-1, AMD3100 (Sigma Aldrich) was added to the soft agar culture medium. When mCherry-tagged MCF7 cells and HMFs were co-cultured, the colonies in soft agar were examined by fluorescence microscopy on day 9. The invasion assay was performed using the BD BioCoat Matrigel Invasion Chamber (BD Biosciences). After transfection with siRNA, HMFs (1 × 10^4^ cells/well) were cultured for 3 days and then a Matrigel insert, loaded with 2.5 × 10^4^ MCF7 cells, was placed in the well. In this setup, the medium for HMFs underneath the insert was the source of the chemoattractants. After 2 days, the noninvading cells on the top of the membrane were removed by scrubbing with a cotton-tipped swab, while the invaded cells on the opposite side were stained with Diff-Quik (TECHLAB). Invaded cells from 5 different fields of each membrane were counted under a light microscope with a 40× objective.

### Immunoblotting and protein quantification

Cell lysate preparation, immunoblotting, and protein quantification were done as previously described [8]. The antibody against α-SMA (1:1,000) was from Dako. The antibodies for vimentin (1:1,000) and N-cadherin (1:1,000) were from Cell Signaling. The secondary antibodies were fluorescently-conjugated IRDye 680 and IRDye 800 from LI-COR Biosciences (1:20,000). Proteins on the membrane were visualized and quantified by the Odyssey infrared imaging system (LI-COR Biosciences).

### Semiquantitative RT-PCR

Total RNA was extracted using the RNeasy Mini Kit (Qiagen), and the cDNAs were generated using the SuperScript First-Strand Synthesis System (Invitrogen). The cycle number was adjusted to allow detection within the linear range of product amplification. The forward and reverse primers for the SDF-1 and actin genes were (5′ to 3′): TGAGAGCTCGCTTTGAGTGA and CACCAGGACCTTCTGTGGAT, and GTGGGGCGCCCCAGGCACCA and CTCCTTAATGTCACGCACGATTTC.

### Acquisition of human tissues

The samples used in this study are anonymized tissues collected between 2000 and 2012 from several sites in the United States and Europe by companies that specialize in tissue acquisition. Pathologists at the hospitals where the tissues were collected performed gross examination to set aside enough tissue for diagnostic purposes. The pathologists then cut the remaining tumor tissue in half, flash-froze half in liquid nitrogen and fixed the other half in formalin, to be embedded in paraffin later. The staff pathologists at the tissue acquisition companies then performed quality control to determine cellularity and to confirm the histology. Because these samples are anonymized, no clinical follow-up is possible. The Institutional Review Board at Baylor College of Medicine determined that these samples are exempt from review.

### Immunohistochemistry and data analysis

We first used cell lines with different Int6 levels to optimize the IHC protocol. These cells were lifted by Versene (GIBCO), and fixed with 10% neutral buffered formalin for 2 hours. The cell pellet was solidified in 4% molten agar (Sigma) and then put in a tissue cassette before finally being embedded in paraffin. Prior to staining, 3-μm sections were cut and deparaffinized by xylenes and a graded alcohol series. Antigen retrieval was performed in 10 mM citrate buffer (pH = 6) in a pressurized cooker with heating (90°C). The sample was then washed and suspended in Tris-Buffered Saline and Tween 20, and 3% H_2_O_2_ was added to block endogenous peroxidases. Two antibodies against Int6 were tested, one of which was described previously [8], and one of which was from Sigma. Although both antibodies worked well, the signal-to-noise ratio is better with the former, so it was used throughout this study (1:200 dilution with cell pellet and 1:50 dilution with tissues; all incubation at room temperature). A negative control was created by treating the same set of samples with non-immune antibody (Dako) matched for species, type, isotype, and concentration. Peroxidase conjugation was performed using the Envision + HRP labeled Polymer Kit from Dako, and diaminobenzidine (DAB+, Dako) was the chromogen. The samples were also counterstained with Harris Hematoxylin.

We selected 20 formalin-fixed, paraffin-embedded human breast tumors that contained “normal” stroma, defined as regions that are at least 2 mm away from the tumor, and then evaluated Int6 levels by IHC in many fibroblasts (57–255) from each region in each sample. These samples were scored by two pathologists independently. Histoscore (possible values 0–300) was computed as the product of the percentage of fibroblasts (possible values 0–100) that scored positive for Int6, and the intensity of Int6 staining in fibroblasts (possible values 0–3). Differences in fibroblast Int6 histoscore and intensity between the normal and tumor regions were assessed by the Wilcoxon signed rank test.

### Statistical analysis

For general quantification of protein intensity and cell growth, values are shown as averages ± SEM. Unpaired student *t* tests were performed to obtain p values.

### Microscopy

Cells were seeded and grown on poly-L-lysine–coated coverslips (BD Biosciences) and later fixed in 3.7% paraformaldehyde, followed by permeabilization in 0.1% Triton X-100. Antibodies against α-SMA (1A4, Dako) and tubulin (Cell Signaling) were used after a 1:200 dilution (2 h at room temperature). The secondary antibody was either conjugated Alexa Fluor 488 or Alexa Fluor 594 from Invitrogen (1:250, 1 h at room temperature). The samples were mounted in Vectashield with DAPI (Vector). Images were captured using an Olympus IX70 microscope via a 60×/1.4 oil objective and deconvolved (constrained iterative) by Slidebook software (Intelligent Imaging Innovations) from a stack of 12 images collected at 0.5-μm intervals. To mark MCF7 cells with mCherry, we constructed the pMCherry vector by replacing the coding sequence of GFP in pEGFP-C1 (Clontech), which carries a neomycin selectable marker, with the coding sequence of mCherry. Subconfluent MCF7 cells were transfected and selected by neomycin. mCherry expression in these cells was confirmed by microscopy.

## Abbreviations

MMTV: Mouse mammary tumor virus
HMF: Human mammary fibroblast
CAF: Carcinoma-associated fibroblast
DCIS: Ductal carcinoma in situ
MSC: Mesenchymal stem cell
SDF-1: Stromal cell-derived factor 1
SMA: Smooth muscle actin
MMT: Mesenchymal-mesenchymal transition
IHC: Immunohistochemistry
ELISA: Enzyme-linked immunosorbent assay
